# Method for plasmid-based antibiotic-free fermentation

**DOI:** 10.1186/s12934-023-02291-z

**Published:** 2024-01-11

**Authors:** Katherine E. Brechun, Marion Förschle, Marlen Schmidt, Harald Kranz

**Affiliations:** Gen-H Genetic Engineering Heidelberg GmbH, Im Neuenheimer Feld 584, 69120 Heidelberg, Germany

**Keywords:** *Escherichia coli*, Antibiotic-free, Plasmid maintenance, Genome engineering, Complementation, Fermentation, Antimicrobial resistance, Antibiotic resistance genes

## Abstract

**Background:**

Antibiotic-based plasmid selection and maintenance is a core tool in molecular biology; however, while convenient, this strategy has numerous drawbacks for biological manufacturing. Overuse of antibiotics and antibiotic resistance genes (ARG) contributes to the development of antimicrobial resistance, which is a growing threat to modern medicine. Antibiotics themselves are costly and therefore often omitted in fermentations, leading to plasmid loss and a corresponding loss in product yield. Furthermore, constitutive expression of a plasmid-encoded antibiotic resistance gene imposes a significant metabolic burden on the cells. For many fermentation products (e.g., in nutrition and medicine), the use of antibiotic resistance genes is subject to strict regulations and should be avoided. We present a method for plasmid selection and maintenance with stringent selection pressure that is independent of antibiotics and ARG. Furthermore, it can be used without any restrictions regarding culture medium and temperature.

**Results:**

The developed method involves modification of a bacterial strain such that an essential gene is expressed genomically under the control of an inducible promoter. A copy of the same essential gene with the endogenous promoter is supplied on a plasmid for selection. In the absence of the inducer for the genomic copy of the essential gene, cells rely on expression of the plasmid-encoded gene copy, leading to tight selection for plasmid maintenance. Induction of the genomic copy of the essential gene enables the engineered strain to be propagated in the absence of a plasmid. Here, we describe the genetic setup and demonstrate long-term, tight selection for plasmid maintenance with a variety of different plasmids and *E. coli* strains.

**Conclusions:**

This method facilitates plasmid-based fermentations by eliminating the need for antibiotic selection and improving plasmid maintenance.

**Supplementary Information:**

The online version contains supplementary material available at 10.1186/s12934-023-02291-z.

## Background

Microbial fermentation provides the means to manufacture an ever-increasing list of commercially relevant molecules typically under benign, environmentally friendly conditions. While the growing white biotechnology industry is posed to play an important role providing sustainable manufacturing solutions, a problematic aspect associated with microbial fermentation is the common reliance on antibiotics and antibiotic resistance genes (ARG) for plasmid selection and maintenance. Antibiotic use in industrial biotechnology is being viewed with increasing concern due to the rapid development of antimicrobial resistance (AMR). The World Health Organization (WHO) has declared AMR poses a profound threat to human health and unless mitigated, may become the leading cause of death by 2050 [1]. The drive to decrease antibiotic use wherever possible is expected to increase the stringency of regulations surrounding antibiotic use in biomanufacturing.

Currently, antibiotic use in biomanufacturing is subjected to a complicated regulatory framework where, depending on the product sector and local legislature, use is strongly discouraged or prohibited. For example, food and feed products derived from genetically modified microorganisms (GMM) in the European Union must undergo a risk assessment to gain market access, which considers the presence and type of any ARG in the process [2]. Approved processes are subject to legislature ensuring the final product is free from GMM and recombinant DNA. However, accidental contamination can occur, most notably, with the discovery of live, multi-resistant *Bacillus subtilis* in a preparation of vitamin B2 [3, 4]. With the development of increasingly sensitive DNA detection methods, analysis of commercialized products has led to the detection of ARG derived from production strains [3, 5], prompting discussion about the feasibility and the relevance of eliminating all microbial DNA from a purified product [6].

In addition to regulatory pressures, the use of antibiotics in a full manufacturing-scale process is often not economically viable, leading most manufacturers to omit antibiotics whenever possible, despite the risk of enriching plasmid-free cells and a corresponding loss in yield. When antibiotic selection is indispensable, expensive process validation of antibiotic removal is required to ensure the final product is free of residual antibiotics. Furthermore, large quantities of antibiotic-contaminated wastewater are produced. Wastewater treatment facilities are not equipped to eliminate chemically diverse synthetic organic compounds, leading to substantial discharges unless there are highly advanced wastewater pre-treatment systems in place [7, 8]. Sub-lethal concentrations of antibiotics in wastewater treatment facilities can lead to enrichment of AMR [8], highlighting the importance of monitoring antibiotics in wastewaters to address the problem. Surveys have found high concentrations of synthetic organic compounds in environmental samples, notably in water samples located close to important biotechnology hubs [9]. In light of these concerns and shortcomings, there has been a call for the fermentation industry to phase out the use of strains containing ARG [6].

Alternative strategies are available for antibiotic-free protein expression. Methods have been developed to integrate a gene of interest into the bacterial chromosome [10, 11]. This allows stable gene expression without the use of an ARG as a selection marker. However, in applications where a higher gene dose is advantageous, protein expression from a multi-copy plasmid is more attractive. Several antibiotic-free plasmid maintenance systems have been developed; in general, these methods can be classified as post-segregational killing strategies or complementation-based techniques.

Post-segregational killing is a naturally occurring bacterial plasmid addiction mechanism, composed of a stable toxin and an unstable antidote that can neutralize the toxin or prevent its synthesis. While in the native system both the toxin and antidote are encoded on the plasmid, it was shown that a toxin-antidote pair can be used for plasmid selection and maintenance by integrating the toxin gene in the chromosome and supplying the antidote gene on a plasmid [12]. Since then, numerous toxin-antidote pairs have been used for plasmid maintenance (reviewed in [13]), including notably the commercialized STABY® system, which uses the *ccd*B/*ccd*A pair [14, 15]. A drawback of this strategy is that the strain expressing the toxin cannot be propagated without a plasmid encoding the neutralizing factor, which means that new host-plasmid combinations require repetitive genome engineering or use of a temperature-sensitive helper plasmid. A synthetic post-segregational killing system avoids this problem by using a negative selection marker that is innocuous under most growth conditions; the RNA-OUT system uses a plasmid-encoded antisense-RNA to inhibit expression of a chromosomally integrated *sac*B gene, which catalyzes formation of a toxic metabolite in the presence of sucrose [16]. The RNA-OUT system carries numerous patents and is used to produce antibiotic-free DNA vaccine vectors commercially.

Complementation offers another option for antibiotic-free plasmid maintenance. Complementation requires a strain lacking a functional copy of an essential gene, allowing gene function to be rescued with a plasmid-encoded gene copy or, if possible, using permissive growth conditions (e.g., supplemented media). Various biosynthetic genes have been used to complement auxotrophic *E. coli* strains, including *dap*D [17], *gly*A [18], *pro*BA [19], and *leu*ABCD [20]. Selection via auxotrophic markers typically requires using defined media to avoid scavenging of the selection metabolite. This restriction is unattractive for large-scale fermentation as the fermentation broth cost can limit economic feasibility. Furthermore, the ability for cells to scavenge a missing metabolite from the media makes the system vulnerable to cross-feeding from lysed cells. This can be avoided by creating a non-feedable auxotroph by targeting a protein with an intracellular role [21–25]. However, the inability to rescue cells through permissive growth conditions means new host-plasmid combinations require repetitive genome engineering or support from helper plasmids. To avoid this ‘locked’ genotype, several strategies have been developed to externally control expression of a targeted essential gene to conditionally impose selection pressure. For example, a repressor-titration system was developed by replacing an endogenous essential gene with an ectopic gene copy under the control of a lac promoter [26]. In the resulting strain, expression of the essential gene was repressed by the lac repressor (*lac*I), unless the strain was supplemented with an inducer (e.g., IPTG) or transformed with a plasmid containing the lac operator site (*lac*O) in molar excess. While this strategy creates non-feedable, conditional complementation, it is incompatible with all lac-based expression platforms and requires tuning to ensure the number of plasmid-encoded operator sites is sufficient to prevent binding of the repressor to the promoter of the essential gene. In a further example, Pagni et al. [27] created a tuneable, non-feedable auxotroph by placing a temperature-responsive transcriptional regulator from *Listeria monocytogenes* in the upstream non-coding region of the essential gene *inf*A. This regulator sequence forms a hairpin structure in the mRNA at 30 °C that occludes the ribosomal binding site and start codon, leading to temperature-dependent inhibition of translation. As a result, a plasmid copy of the essential gene can be used for complementation; however, strict plasmid maintenance is only achieved at lower temperatures, which is a severe limitation for industrial fermentation.

To add to this toolkit of antibiotic-free plasmid expression platforms, we created a conditional, non-feedable auxotroph by placing an essential gene under the control of an inducible promoter, such that the strain can be rescued either by supplying a plasmid-encoded copy of the essential gene or via supplementation with the inducer. Importantly, this strategy relies on positive selection pressure, which is more robust than negative selection, as negative selection agents (e.g., toxins) can be diffused by any mutation terminating expression. As a selection marker, we chose to regulate expression of the essential gene *inf*A, encoding Translation Initiation Factor 1 (IF1). The gene *inf*A is an interesting choice for a selection marker because it is small (72 residues) and essential under all culture conditions, allowing selection to be imposed regardless of media choice. In fact, complementation using *inf*A has been used on a few occasions for plasmid maintenance [21–23, 27], and the characterization of these systems has demonstrated the suitability for plasmid selection. For example, it has been shown that released IF1 is not absorbed by other cells, eliminating the concern of cross-feeding [21], and others have demonstrated that adjusting the *inf*A expression level on a complementation plasmid can tune plasmid copy numbers with minimal cell-to-cell variation [23]. However, these *inf*A-based selection mechanisms have not received wide-spread adoption, likely due to associated temperature restrictions [27] (see above) or cumbersome handing [21–23]. In references [21–23], complementation was achieved using a simple chromosomal deletion of *inf*A, and therefore repetitive strain engineering or helper plasmids are required to transform the engineered auxotrophic strain with a new plasmid. We present a novel strategy that is temperature independent and overcomes the inconvenient ‘locked’ strain-plasmid genotype by controlling chromosomal *inf*A expression using the arabinose promoter. As a result, arabinose supplementation enables propagation of the ‘empty’ strain, while the absence of arabinose induces selection pressure for an *inf*A-encoding plasmid. The arabinose promoter has been shown to exhibit tight control of protein expression and has been used for other highly sensitive expression studies [28], making it an ideal choice for this system.

Here, we describe the development of the *inf*A-selection strains and demonstrate long-term plasmid maintenance. We show remarkable robustness of the strategy, with stable maintenance of plasmids from different plasmid classes in a variety of *E. coli* strains.

## Results and discussion

### Development of strain with a conditionally expressed essential gene

**Fig. 1:**
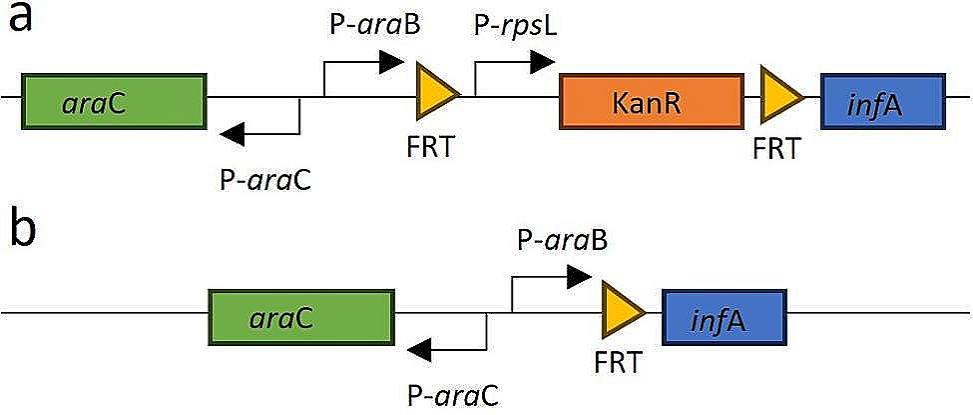
Schematic representation of the genome modifications to yield arabinose-inducible *inf*A expression. **a** In the first step, the arabinose repressor, an arabinose-inducible promoter and an FRT-flanked constitutively expressed selection marker cassette were integrated upstream of the *inf*A coding sequence to replace the endogenous *inf*A promoter. **b** Flp recombinase-mediated excision of the selection marker and its constitutive promoter leads to arabinose-dependent expression of *inf*A. *ara*C, arabinose repressor; P-*ara*C, *ara*C promoter; P-*ara*B, ribulokinase promoter (arabinose-inducible); FRT, flippase recognition target; P-*rps*L, promoter from 30 S ribosomal subunit protein S12 (constitutive); Kan^R^, kanamycin resistance gene (aminoglycoside phosphotransferase); *inf*A, Translation Initiation Factor 1

To develop an *inf*A-selection strain, the endogenous *inf*A promoter was replaced in the *E. coli* K-12 strain HS996 using λ-red recombineering in a two-step protocol [11]. First, a cassette containing the arabinose repressor (*ara*C), an arabinose-inducible promoter and an FRT-flanked (flippase recognition target), constitutively expressed kanamycin resistance gene (Kan^R^) was integrated into the genome to replace the endogenous *inf*A promoter (Fig. 1a). As a result of the integration, *inf*A was expressed from the constitutive promoter driving Kan^R^ expression as the second gene in an operon. In a second step, the FRT-flanked selection cassette was removed using Flp recombinase. At this point, the culture media was supplemented with arabinose (0.25%). Excision of the FRT-flanked sequence placed the arabinose promoter upstream of the *inf*A gene, leading to arabinose-inducible *inf*A expression (Fig. 1b). This strain is referred to as HS996-*inf*A.

### Antibiotic-free transformation and long-term plasmid stability

**Fig. 2:**
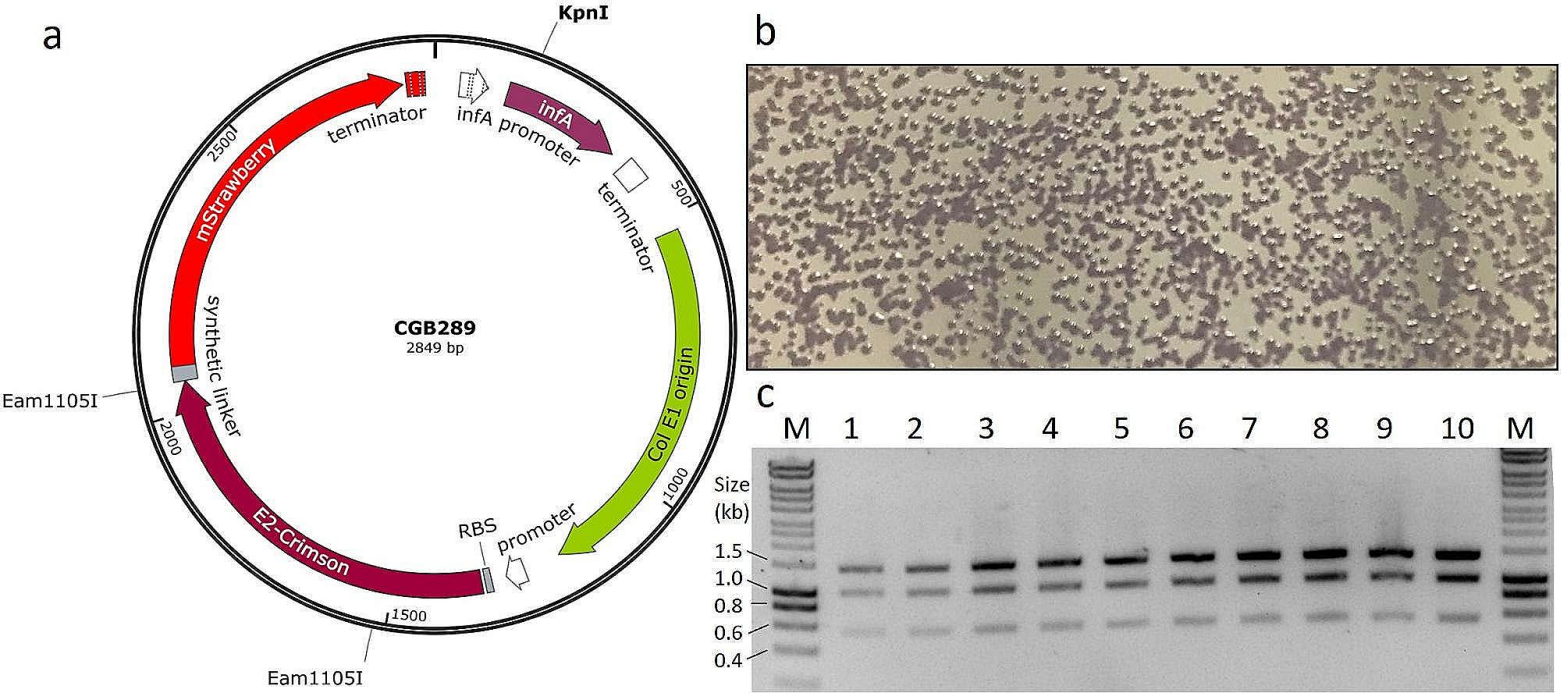
Antibiotic-free transformation. **a** Plasmid map of CGB289 showing the location of the restriction enzyme sites. **b** The engineered strain *E. coli* HS996-*inf*A was transformed using *inf*A selection with the plasmid CGB289. The purple pigmentation of the resulting colonies indicates that all colonies have taken up plasmid. **c** Ten isolated colonies were selected from the transformation plate and analyzed with a restriction digest using KpnI and Eam1105I. All samples displayed the expected banding pattern: 1353 bp, 965 bp and 531 bp

Next, the engineered strain *E. coli* HS996-*inf*A was transformed with a plasmid containing a constitutively expressed *inf*A gene as a selection marker. The *inf*A-plasmid was assembled using a three-piece Golden Gate [29] reaction. The three fragments consisted of (i) the colE1 origin of replication, (ii) the coding sequence for a chromogenic marker protein (two chromoproteins, E2-Crimson and mStrawberry [30], genetically fused with a short synthetic linker) expressed with a constitutive promoter, and (iii) an *inf*A expression cassette including its endogenous promoter (amplified from the *E. coli* HS996 genome) and a *Not*I-flanked chloramphenicol resistance (Cm^R^) cassette (Fig. 2a). We were careful not to include passenger genomic sequence upstream of the *inf*A promoter to prevent wild-type promoter reversions; while both the plasmid and the genome contain the same wild-type *inf*A coding sequence, there is no other homology present on the plasmid that could spur a recombination event to bring the endogenous *inf*A promoter back into the genome. The assembled Golden Gate product was transformed into *E. coli* HS996 using chloramphenicol (Cm) selection. This plasmid was designated CGB285 (see plasmid list, Table 1). The Cm^R^ gene was subsequently removed by digesting with NotI and religating the plasmid. The resulting plasmid containing only *inf*A for selection was designated CGB289. The chromoprotein gene was included on these plasmids so that plasmid presence could be visually assessed for demonstrative purposes.

**Table 1:** Plasmids developed and used in this study

Name	Features	Description
CGB285	colE1-*inf*A-chromoprotein-Cm	High copy, *inf*A selection and Cm selection, chromoprotein expression
CGB288	p15A-*inf*A-chromoprotein	Low copy, *inf*A selection, chromoprotein expression
CGB289	colE1-*inf*A-chromoprotein	High copy, *inf*A selection, chromoprotein expression
CGB290	R6K-*inf*A-chromoprotein	Medium copy, *inf*A selection, chromoprotein expression
CGB237	pQE80L-TNFα-*inf*A -Cm	High copy, *inf*A selection and Cm selection, TNFα expression from the T7 promoter

Transformation of the modified HS996-*inf*A strain using *inf*A-based selection was performed using a typical transformation protocol with minor modifications. First, during competent cell generation, culture media was supplemented with 0.25% arabinose to induce expression of the genomic *inf*A copy. After transformation, cells were plated on LB-agar without arabinose to select for cells with plasmid. Transformation of HS996-*inf*A with the plasmid CGB289 yielded purple colonies, indicating all colonies contained plasmid DNA and expressed the chromogenic marker protein (Fig. 2b). Ten colonies were selected for further analysis. The colonies were inoculated in LB-media without arabinose and after overnight growth, plasmid DNA was isolated and analyzed with a restriction digest. As expected, all cultures contained plasmid DNA with the correct digestion pattern (Fig. 2c). We conclude that *inf*A-based complementation provides effective plasmid selection; the lack of plasmid-free colonies after transformation indicates that single cells without plasmid are unable to proliferate and form colonies on arabinose-free media.

**Fig. 3:**
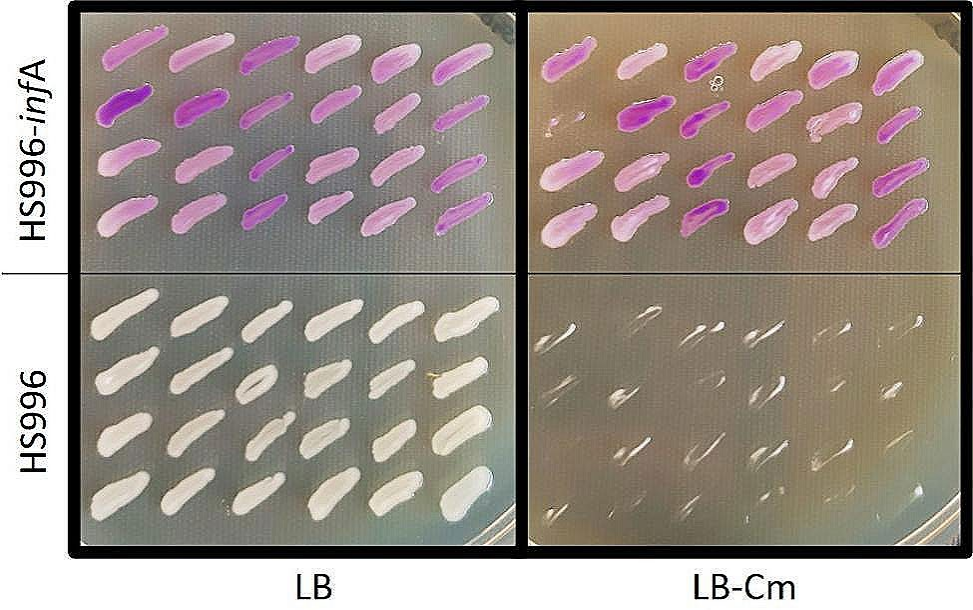
Plasmid maintenance after a week of growth in media without antibiotics. The engineered strain HS996-*inf*A showed complete plasmid maintenance after growth in LB-media without antibiotic supplementation, as shown by the colony colour and growth on the LB-Cm plate. All isolated HS996 colonies were found to have lost the same plasmid after a week of growth in non-selective conditions, as shown by the lack of pigment and susceptibility to Cm

We next tested if this method enables long-term plasmid maintenance. For this, the engineered strain HS996-*inf*A was transformed with the plasmid CGB285, containing the coding sequence for a chromogenic marker, *inf*A and Cm^R^. The transformation was performed as described above without using antibiotic selection. In parallel, non-modified HS996 was transformed with the same plasmid using Cm selection. An isolated colony from each transformation was inoculated into LB-medium without supplementation, and the cultures were incubated with daily passaging into fresh, non-supplemented LB-medium for 5 days. After this, the cultures were streaked on LB-agar plates to obtain isolated colonies. Following incubation, 24 colonies from each strain were patched onto two plates: LB-agar and LB-agar-Cm (Fig. 3). All HS996-*inf*A colonies examined maintained the plasmid, as indicated by the purple pigment of the cultures and resistance to Cm. A single patch showed no growth on the Cm plate despite a strong purple colour on the corresponding patch on the LB plate; further investigation showed that the transposable element IS1 had been inserted into the Cm^R^ gene, leading to Cm sensitivity. In contrast, none of the HS996 colonies examined maintained the plasmid. The complete loss of plasmid DNA was somewhat surprising because CGB285 contains a high-copy origin of replication (colE1); however, it has previously been noted that overexpression of a chromoprotein exerts a high fitness cost on the host, leading to strong selection pressure for loss of expression [30].

### Fitness of the engineered strain

**Fig. 4:**
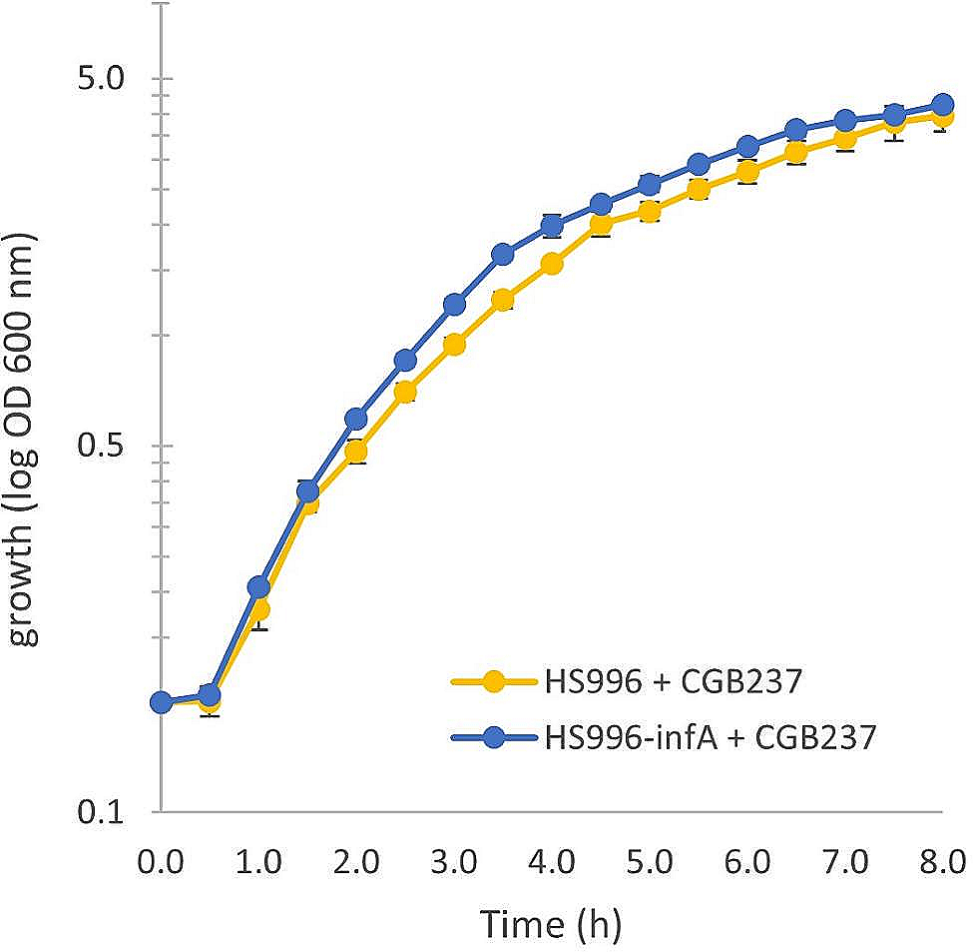
Growth curves comparing the growth of wild-type HS996 with the growth of the modified strain, HS996-*inf*A. Each strain contains the plasmid CGB237 and was grown with conditions imposing plasmid selection pressure (i.e., HS996 in LB-Cm and HS996-*inf*A in LB). Error bars show variation from three biological replicates

Next, we examined the fitness of the engineered strain, HS996-*inf*A. A potential concern with this strategy is that relocation of a gene central to protein synthesis onto a plasmid could significantly compromise strain fitness. In this regard, it should be noted that subjecting cells to antibiotic stress and overexpressing a foreign antibiotic resistance gene is also a significant intervention into *E. coli* metabolism. Indeed, certain ARG are known to reduce *E. coli* fitness [31], and it has previously been shown that expression of an antibiotic resistance gene (which occurs regardless of whether cultures are supplemented with the antibiotic or not) subjects cells to a high metabolic burden that can lead to a reduced yield of a fermented product [15, 26]. To examine the possibility of reduced strain fitness, the growth rate of the engineered strain, HS996-*inf*A was compared with that of the parental strain, HS996. Since cultures containing the chromoprotein plasmid showed a tendency to vary in pigment intensity (see Fig. 3), suggesting non-uniform chromoprotein expression, a new *inf*A-plasmid was developed to ensure a uniform metabolic load during growth experiments. The new plasmid, termed CGB237, encodes *inf*A, Cm^R^ and an expression cassette with an inducible model protein (SI Fig. 1). The strains HS996-*inf*A and HS996 were transformed with CGB237. In the modified strain, the transformation was performed using *inf*A selection (i.e., by not supplementing plates with arabinose), while in the parental strain, the transformation was performed using Cm selection. Growth curves were measured for each strain in LB-media (supplemented with Cm for the parental strain to ensure plasmid maintenance) using biological triplicates (Fig. 4). The specific growth rate of the modified strain (0.78 +/-0.05 h^− 1^) was slightly faster than that of the parental strain (0.66 +/- 0.05 h^− 1^), indicating that *inf*A complementation could offer an improvement in cell fitness compared to antibiotic-based selection.

### Robustness of the strategy

We next investigated the robustness of this selection method. We first tested *inf*A selection using plasmids with different copy numbers in the engineered strain HS996-*inf*A. Then, we modified different *E. coli* strains to create the inducible-*inf*A genotype in different *E. coli* backgrounds.

**Fig. 5:**
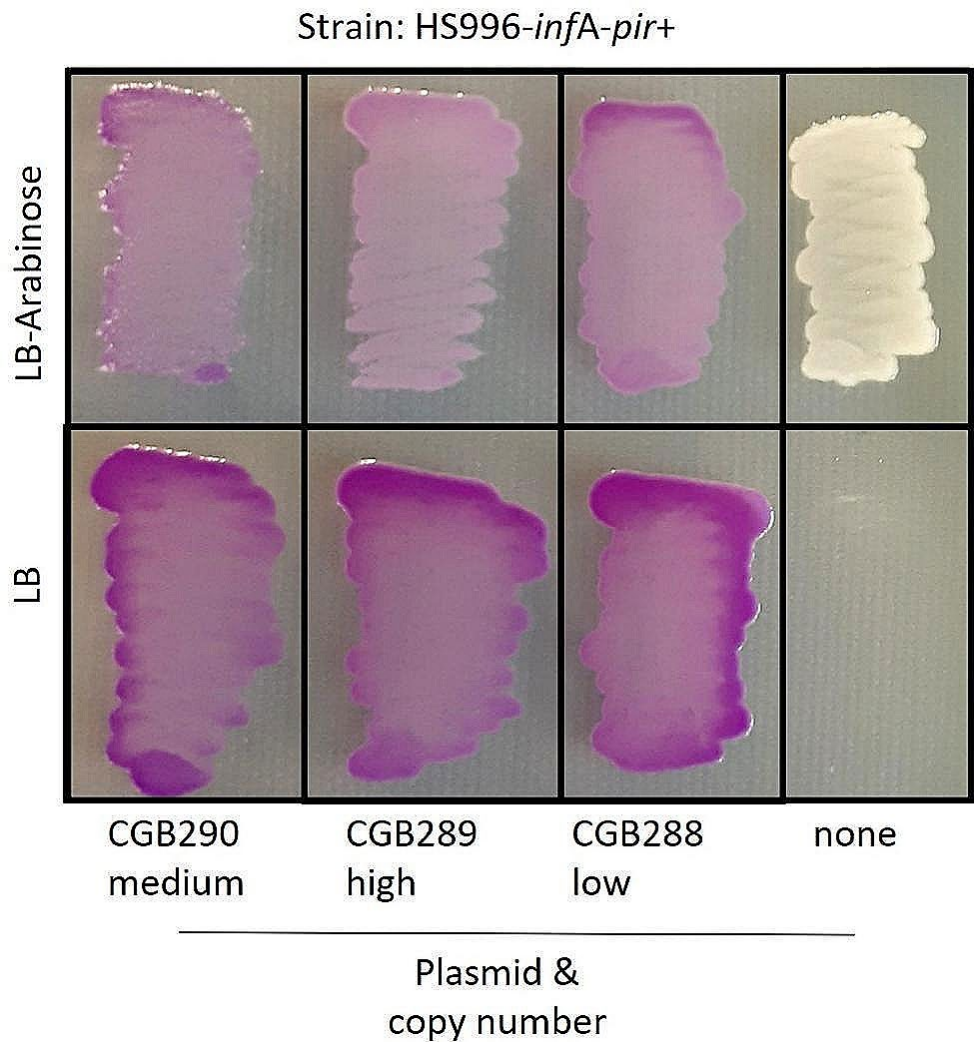
*inf*A-based plasmid selection using different plasmid replication origins. The strain HS996-*inf*A-*pir +* was transformed with plasmids differing only in the origin of replication, and subsequently streaked on LB-agar with and without arabinose supplementation. The empty strain, without an *infA* plasmid, showed no growth in the absence of arabinose. The strains with plasmid grew on both plates

To determine if the plasmid copy number influences the selection mechanism, plasmids were assembled with the *inf*A expression cassette, a chromoprotein expression cassette and different origins of replication. These plasmids do not contain an antibiotic selection marker. Three different replication sequences were tested (p15A, ColE1 and R6K), covering plasmid copy numbers of roughly 10–100 copies per cell. In order to test the R6K plasmid, we further modified the strain HS996-*inf*A by integrating the *pir* gene, which is essential for R6K plasmid replication [32], into the *umu*C locus as described in the methods section. The resulting strain HS996-*inf*A-*pir +* was transformed with each of the three plasmids, and each strain was subsequently streaked on both an LB-agar and an LB-agar-arabinose plate. The engineered strain without a plasmid only grew on the plate supplemented with arabinose; all three transformed strains grew on both plates and were purple, showing presence of the chromoprotein-expressing plasmid (Fig. 5).

It is worth highlighting that the combination of the R6K origin of replication and the *inf*A selection marker yields a plasmid with a backbone of approximately 800 bases. This small, antibiotic-free backbone could be particularly valuable for DNA production in gene therapy; plasmids used in gene therapy need to be ARG-free and the plasmid backbone DNA should be minimized because bacterial DNA sequences may contain immunostimulatory motifs that can lead to gene silencing [33].

**Fig. 6:**
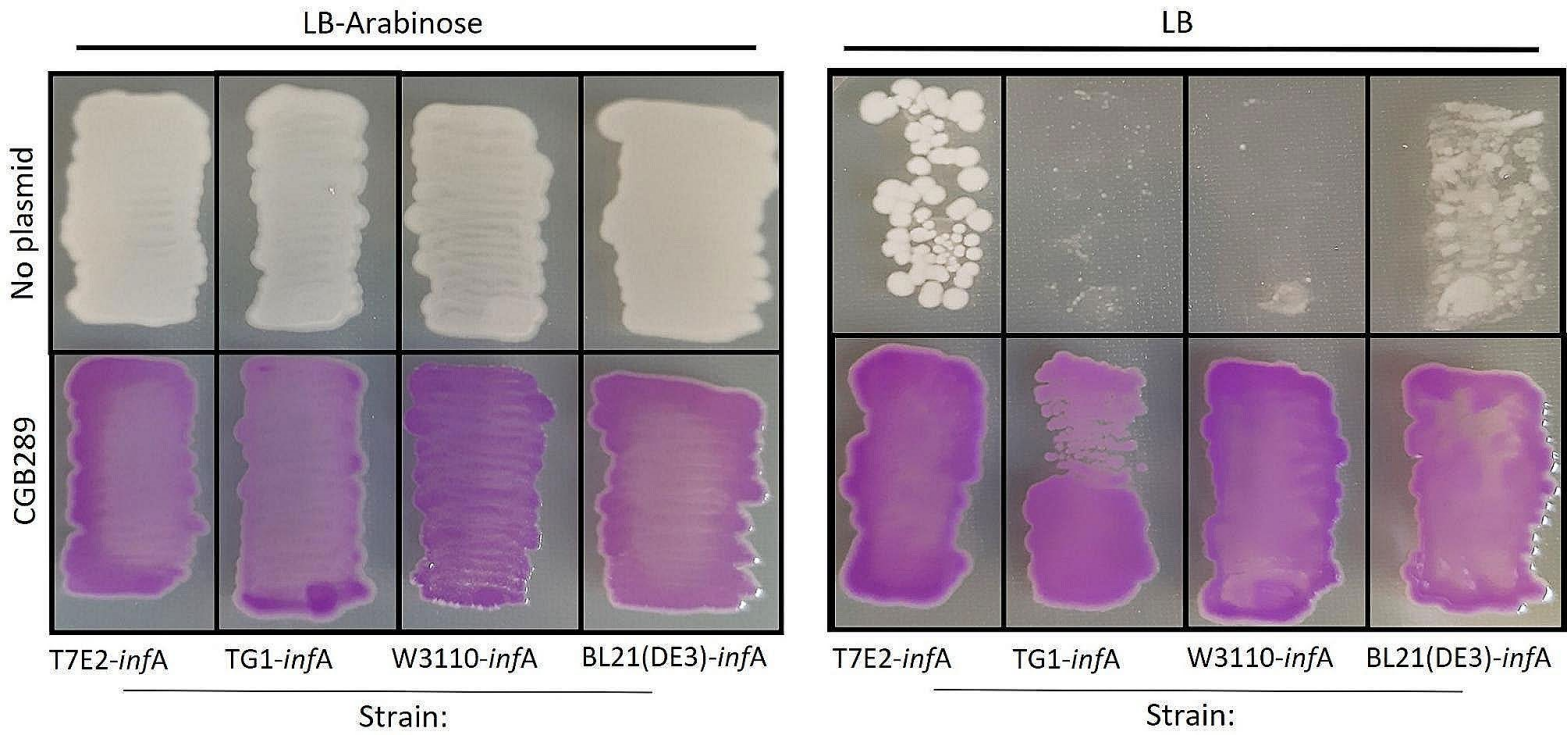
*inf*A-based plasmid selection in different *E. coli* strain backgrounds. The strains were transformed with the plasmid CGB289, and subsequently streaked on LB-agar with and without arabinose supplementation. The empty strains showed no growth or significantly reduced growth in the absence of arabinose. The strains with plasmid grew on both plates and showed purple pigment from the plasmid-encoded chromoprotein

Next, we tested the *inf*A selection method in different *E. coli* backgrounds. Testing different *E. coli* strains is important because it has been found previously that selection mechanisms can be strain dependent [34]. For example, post-segregational killing systems based on *ccd*B/*ccd*A would be expected to be less efficient in strains with an F plasmid (which naturally contains a *ccd*B/*ccd*A plasmid addiction system) and in strains devoid of Lon protease (as Lon is required to ensure rapid turnover of the CcdA protein antidote) [35]. To determine if the *inf*A selection mechanism is functional in other *E. coli* strains, the *inf*A promoter was exchanged with the arabinose promoter in two B-strains (*E. coli* BL21(DE3) and T7E2), and in two K12-strains (*E. coli* W3110 and TG1) using the procedure described for the generation of HS996-*inf*A. Successfully modified strains were obtained in all cases. Each of the modified strains was transformed with CGB289 using LB without arabinose to select for plasmid uptake. Analogous to the experiment in Fig. 5, each strain was streaked on both an LB-agar and an LB-agar-arabinose plate. All transformed strains grew on both plates and were purple; the engineered strains without plasmid showed minimal residual growth or no grow on the plate without arabinose (Fig. 6).

The residual growth is insignificant compared to the growth of the strains containing plasmid. Therefore, loss of the plasmid-encoded *inf*A copy creates a strong growth disadvantage that would prevent a plasmid-free population from proliferating in a fermentation. This contrasts with traditional antibiotic-based plasmid maintenance; when antibiotic supplementation is omitted, cells without plasmid gain a significant growth advantage by eliminating the metabolic burden imposed by plasmid replication and gene expression (of both the gene of interest and the antibiotic resistance gene).

### Antibiotic-free protein expression

**Fig. 7:**
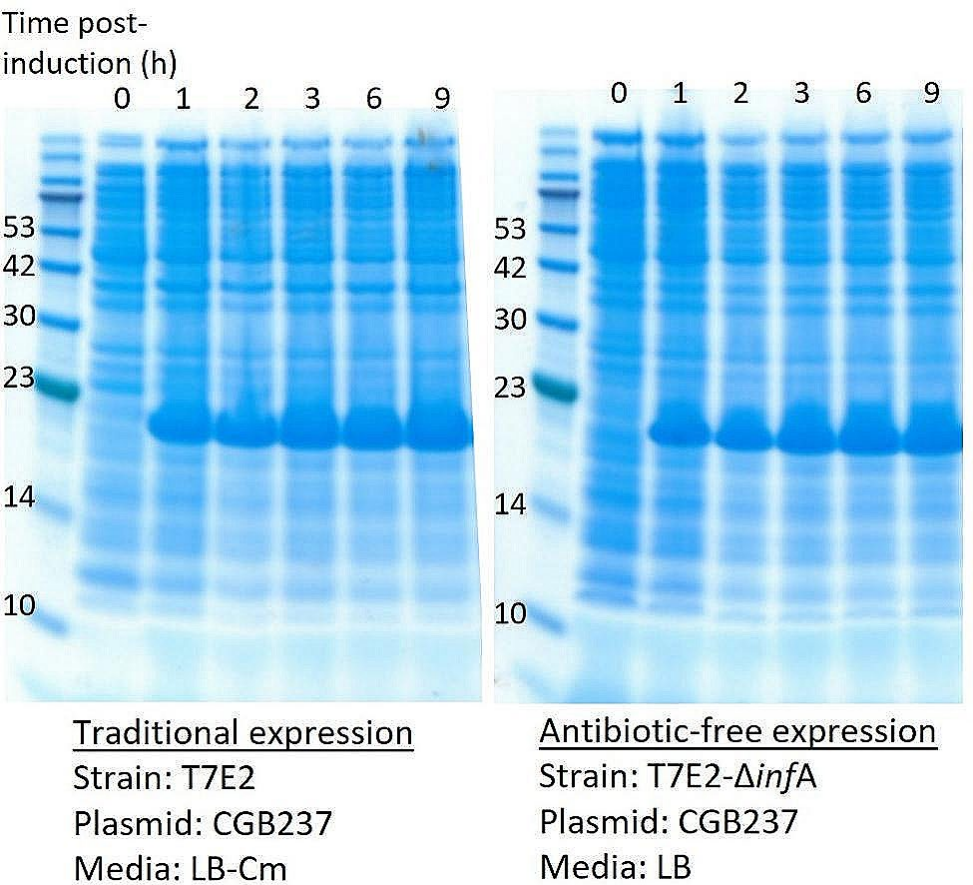
Protein expression using antibiotic-based plasmid maintenance versus *inf*A-based plasmid maintenance as shown by full cell lysates after induction of protein expression. **a** SDS PAGE showing samples obtained from a traditional protein expression culture using antibiotic-based plasmid maintenance. **b** SDS PAGE showing samples obtained from an antibiotic-free expression culture using *inf*A complementation for plasmid maintenance. The model protein TNFα is 18.8 kDa. The protein marker (PM2500 ExcelBand™, SMOBIO Technology) is shown with the fragment sizes annotated on the gel in kDa

Finally, as a proof-of-principle, we tested protein expression using *inf*A-based plasmid maintenance in comparison with traditional antibiotic-based selection pressure. For this experiment, we used the plasmid CGB237, which encodes *inf*A, Cm^R^ and an inducible expression cassette for the model protein, tumor necrosis factor alpha (TNFα). As an expression host, we selected the strain T7E2 and the corresponding modified strain, T7E2-*inf*A. T7E2 is a BL21(DE3) derivative that contains the gene for T7 RNA polymerase, but has been cured of the DE3 lysogen [36]. CGB237 was transformed into T7E2-*inf*A using the absence of arabinose for plasmid selection, and into wild-type T7E2 using Cm selection. A single colony from each transformation was inoculated into media with no additives (T7E2-*inf*A) or with Cm (T7E2) for a small-scale expression. During expression, normalized culture samples were taken hourly after induction with IPTG and subsequently analyzed by sodium dodecyl sulfate polyacrylamide gel electrophoresis (SDS PAGE). In both cases, a protein band at approximately 19 kDa was visible 1 h after induction, showing expression of the TNFα model protein (Fig. 7). A high level of protein production was observed in both cultures.

## Conclusions

The advantages of antibiotic-free biomanufacturing are highly attractive. Eliminating antibiotics and ARG from a fermentation process simplifies meeting regulatory requirements and improves the product safety profile, creating a marketable advantage. Moreover, antibiotic-based plasmid maintenance is unsuitable for industry; the prohibitive cost of antibiotic supplementation at industrial scale leads many manufacturers working with antibiotic-based systems to omit antibiotics in production, fermenting completely without plasmid selection pressure, despite the risk of decreased yield. Several antibiotic-free plasmid maintenance methods have been developed previously to address this problem. However, these methods have not received widespread adoption in industry, likely due to restrictions regarding media and temperature use, or due to cumbersome protocols required to transform the engineered strains with a new plasmid.

We present a robust and simple method for plasmid maintenance that eliminates the need for antibiotics and ARG. By placing an essential gene under the control of an inducible promoter, a conditional auxotrophic strain is generated, which requires a plasmid copy of the essential gene in the absence of the inducer. In theory, we expect that this selection strategy could function with any essential gene, or any combination of genes rendered essential due to combinatorial knockouts and/or growth conditions. Furthermore, we expect that this strategy could be extended to other microorganisms, if an appropriate inducible promoter is available. Since the ‘empty’ strain can be propagated by supplementing the culture with an inducer, it is possible to transform the strain with any complementing plasmid without repetitive genome engineering or helper plasmids. Using the expression of an endogenous gene for plasmid selection minimizes the metabolic burden on cells, which should leave more resources available to maximize a product yield. Using the absence of an inducer to impose selection, yields continuous selection pressure for plasmid presence without adding any costs to the production. Finally, this simple selection mechanism is highly flexible, operating independently of temperature and media choice, and is compatible with a wide range of plasmid types and strain backgrounds. We conclude that this conditional auxotrophic strain offers a practical solution to eliminate the use of antibiotics and ARG for routine plasmid selection in biological manufacturing.

## Methods

### Strains

Plasmid assembly was performed in *E. coli* HS996. Strain engineering was performed in numerous *E. coli* strains; please see Table S1 in the supplementary data for a detailed list of the *E. coli* strains used and developed in this study. Parental strains used in this study were grown in LB-media. Strains with a conditionally expressed essential gene were grown in LB-media supplemented with 0.25% arabinose in the absence of plasmid. When indicated, LB-media was supplemented with Cm (at 15 µg/ml for low-copy plasmids and 30 µg/ml for medium- and high-copy plasmids), with Kan (at 15 µg/ml for genomic integration), or with ampicillin (Amp, at 50 µg/ml for low-copy plasmids).

### Genome engineering

Strain engineering was performed using λ-red recombineering using the helper plasmid pSC101-BAD-gbaA, as described in Wang et al. [37]. Recombineering was performed with 200–1,000 ng of linear DNA with 50–200 bp of homology for genome integration. The fragments were generated using standard PCR conditions, digested with DpnI, and then purified using a GeneJET PCR Purification Kit (Thermo Fisher Scientific) and eluted in water. Subsequent marker-excision was performed via flp-mediated recombination, using a temperature-sensitive, low-copy helper plasmid with a temperature-inducible Flp gene. Briefly, the target strain was transformed with the helper plasmid, plated on LB-agar with antibiotics for plasmid selection (Amp) and strain selection (Kan). After overnight incubation at 30 °C, a single colony was grown to saturation in 1.5 mL of LB-Amp-Kan (30 °C with 800 rpm in a temperature-controlled mixing block). Subsequently, the culture was diluted 30-fold in fresh LB without antibiotics and grown for 3 h at 30 °C with 800 rpm. The culture was then incubated at 37 °C for 1 h to induce Flp expression, streaked on an LB-agar plate without antibiotics and incubated overnight at 37 °C. Single colonies were then patched on LB-agar plates to check for Kan sensitivity (excision of the FRT-flanked marker) and Amp sensitivity (loss of the helper plasmid). The strains were sequenced over the FRT-scar to confirm marker excision.

The procedure used to modify the *inf*A promoter is outlined in the results section. The *pir-116* gene was integrated into HS996-*inf*A in the *umu*C locus using a two-step λ-red recombineering protocol. First, a cassette containing the *pir-116* gene with its endogenous promoter and an FRT-flanked Kan^R^ was integrated in the *umu*C locus and recovered using Kan selection pressure (15 µg/ml). Subsequently, the FRT-flanked Kan^R^ was removed using Flp-recombinase, resulting in the strain HS996-*inf*A-*pir*+. All modifications were confirmed with sequencing.

### Plasmid construction and transformation

Plasmids were constructed using λ-red recombineering, Golden Gate assembly [29], and /or restriction enzyme-based cloning. All modifications were confirmed with sequencing. All plasmids used in this study are shown in Table 1.

We routinely use electroporation for transformations. To generate electrocompetent cells, an overnight culture was inoculated into fresh LB-medium and grown to an optical density at 600 nm (OD_600_) of approximately 0.6. The culture was pelleted at 4 °C, washed with ice-cold 10% glycerol twice, and then used immediately for electroporation. For strains with conditional *inf*A expression, the LB-media was supplemented with 0.25% arabinose. Electroporation was performed with cooled cuvettes (1 mm gap) in an Eppendorf Electroporator 2510 with 1350 V. Electroporated cells were rescued in 1 ml of room temperature LB-medium (without arabinose), grown for 1 h at 37 °C with 800 rpm in a temperature-controlled mixing block and plated on an LB-agar plate with plasmid selection pressure (lack of arabinose or antibiotics as required). We found transformations using *inf*A-based selection routinely yielded significantly more colonies, so we reduced the culture volume plated to obtain single colonies.

### Plasmid stability monitoring

To monitor plasmid stability, a single colony of the desired strain was inoculated from the transformation plate into LB-medium without supplementation, and the culture was incubated at 37 °C with agitation (800 rpm in a temperature-controlled mixing block). The culture was passaged into fresh LB-medium once every 24 h for five days. Following this incubation period, the culture was streaked on an LB-agar plate and incubated overnight at 37 °C. Subsequently, 24 isolated colonies were patched onto two plates: one containing non-supplemented LB-agar and the second containing LB-agar with antibiotic (Cm at 30 µg/mL) and incubated overnight at 37 °C. Prior to imaging, the plates were incubated at 4 °C to facilitate maturation of the chromoprotein pigment.

### Growth curves

To compare the growth rate of the strains HS996-*inf*A and HS996, each strain was transformed with the plasmid CGB237 using either the lack of arabinose or Cm (30 µg/ml) respectively for plasmid selection. After incubation, 3 isolated colonies from each strain were each inoculated into 50 mL LB-media with appropriate plasmid selection (lack of arabinose or Cm) and incubated at 37 °C with 200 rpm overnight. The following morning, each culture was inoculated into fresh medium with plasmid selection to a starting OD_600_ of 0.1. The cultures were incubated at 37 °C with 200 rpm and culture samples were taken at regular intervals to monitor turbidity (OD_600_) using an Eppendorf BioPhotometer. The specific growth rate was calculated using the following formula:

$$\mu =\frac{\text{ln}\left(OD2\right)-\text{l}\text{n}\left(OD1\right)}{t2-t1}$$

### Streak plates

To compare strain growth with and without arabinose supplementation, an isolated colony of each strain was inoculated into LB-media and incubated overnight at 37 °C with shaking (800 rpm in a temperature-controlled mixing block). This step was done for all strains, including strains without plasmid, in order to deplete intracellular *inf*A concentrations prior to plating on *inf*A-selective media. The following day, each culture was streaked on an LB-agar and an LB-agar-arabinose (0.25%) plate, and incubated overnight 37 °C. Prior to imaging, the plates were incubated at 4 °C to facilitate maturation of the chromoprotein pigment.

### Protein expression

The strains T7E2-*inf*A and T7E2 were transformed with the plasmid CGB237 using the absence of arabinose or Cm (30 µg/mL) respectively for selection. A single colony from each transformation was inoculated into 10 mL LB-media with appropriate plasmid selection pressure (lack of arabinose or Cm) and incubated overnight at 37 °C with 200 rpm. The following morning, each culture was inoculated into 10 mL fresh medium with appropriate plasmid selection pressure (lack of arabinose or Cm) and incubated at 37 °C with 200 rpm. At an OD_600_ of approximately 0.6, protein expression was induced by adding IPTG to a final concentration of 1 mM. Culture samples were taken hourly upon induction, normalized to a standard OD, and analyzed on an SDS PAGE gel.

### Electronic supplementary material

Below is the link to the electronic supplementary material.

**Table S1**: Genotypes of the E. coli strains used and developed in this study.

SI Fig. 1: Plasmid map of CGB237 used for experiments shown in Fig. 4 and Fig. 7.

## Data Availability

No datasets were generated or analysed during the current study.
