# Supplementary material for: Method for plasmid-based antibiotic-free fermentation
Source: Microb Cell Fact. 2024 Jan 11;23:18. doi: 10.1186/s12934-023-02291-z (PMC10782701; doi:10.1186/s12934-023-02291-z)
Supplement: Supplementary file 1 — Table S1: Genotypes of the E. coli strains used and developed in this study. [file 12934_2023_2291_MOESM1_ESM.docx]

**Table S1:** Genotypes of the *E. coli* strains used and developed in this study.

| Strain | Genotype | Source |
| --- | --- | --- |
| HS996 | F^-^ *mcr*A Δ(*mrr-hsd*RMS-*mcr*BC) φ80*lac*ZΔM15 Δ*lac*X74 *rec*A1 *ara*D139 Δ(*ara-leu*)7697 *gal*U *gal*K *rps*L (Str^R^) *end*A1 *nup*G *fhu*A::IS2 | Gene Bridges GmbH |
| HS996-*inf*A | F^-^ *mcr*A Δ(*mrr-hsd*RMS-*mcr*BC) φ80*lac*ZΔM15 Δ*lac*X74 *rec*A1 *ara*D139 Δ(*ara-leu*)7697 *gal*U *gal*K *rps*L (Str^R^) *end*A1 *nup*G *fhu*A::IS2 ∆P_infA_::*ara*C-P_ara_-FRT-*inf*A | This study |
| HS996-*inf*A-*pir*+ | F^-^ *mcr*A Δ(*mrr-hsd*RMS-*mcr*BC) φ80*lac*ZΔM15 Δ*lac*X74 *rec*A1 *ara*D139 Δ(*ara-leu*)7697 *gal*U *gal*K *rps*L (Str^R^) *end*A1 *nup*G *fhu*A::IS2 ∆P_infA_::araC-P_ara_-FRT-*inf*A Δ*umu*C::P_pir_-pir-116-FRT | This study |
| BL21DE3 | F^-^ *omp*T *gal* *dcm* *lon hsd*S_B_(r_B_^–^m_B_^–^) λ(DE3 [*lac*I *lacUV5*-T7p07 *ind*1 *sam*7 *nin*5]) [*mal*B+]K-12(λS) | BCCM, Belgian Coordinated Collection of Microorganisms |
| BL21DE3-*inf*A | F^-^ *omp*T *gal* *dcm* *lon* *hsd*S_B_(r_B_^–^m_B_^–^) λ(DE3 [*lac*I *lacUV5*-T7p07 *ind*1 *sam*7 *nin*5]) [*mal*B+]K-12(λS) ∆P_infA_::*ara*C-P_ara_-FRT-*inf*A | This study |
| T7E2 | F^-^ *omp*T *gal* *dcm* [*lon*] *rps*L150(Strep^R^) Δ*rac*::*pgl* K-12 *hsd*S_B_(r_B_^–^m_B_^–^) λ(DE3 [*lac*I *lacUV5*-T7 polymerase] Δ*int*-ea59::loxP) | Gen-H Genetic Engineering Heidelberg GmbH |
| T7E2-*inf*A | F^-^ *omp*T *gal* *dcm* [*lon*] *rps*L150(Strep^R^) Δ*rac*::*pgl* K-12 *hsd*S_B_(r_B_^–^m_B_^–^) λ(DE3 [*lac*I *lacUV5*-T7 polymerase] Δ*int*-ea59::loxP) ∆P_infA_::*ara*C-P_ara_-FRT-*inf*A | This study |
| TG1 (derivative) | *sup*E *thi*-1 Δ(*lac*-*pro*AB) Δ(*mcr*B-*hsd*SM)5, (rK-mK-); F' [*tra*D36 *pro*AB+ *lac*I^q^ *lac*ZΔM15], *rha*A- | DSMZ-German Collection of Microorganisms and Cell Cultures GmbH  (DSM 6056) |
| TG1-*inf*A | *sup*E *thi*-1 Δ(*lac*-*pro*AB) Δ(*mcr*B-*hsd*SM)5, (rK-mK-); F' [*tra*D36 *pro*AB+ *lac*I^q^ *lac*ZΔM15], *rha*A- ∆P_infA_::*ara*C-P_ara_-FRT-*inf*A | This study |
| W3110 | F^-^ λ^-^ *ilv*G- *rpo*S(Am) *rfb*-50 *rph*-1 IN(*rrn*D-*rrn*E)1 | Coli Genetic Stock Center (CGSC#4474) |
| W3110-*inf*A | F^-^ λ^-^ *ilv*G- *rpo*S(Am) *rfb*-50 *rph*-1 IN(*rrn*D-*rrn*E)1 ∆P_infA_::*ara*C-P_ara_-FRT-*inf*A | This study |
