# Supplementary figures and images for: Method for plasmid-based antibiotic-free fermentation
Source: Microb Cell Fact. 2024 Jan 11;23:18. doi: 10.1186/s12934-023-02291-z (PMC10782701; doi:10.1186/s12934-023-02291-z)

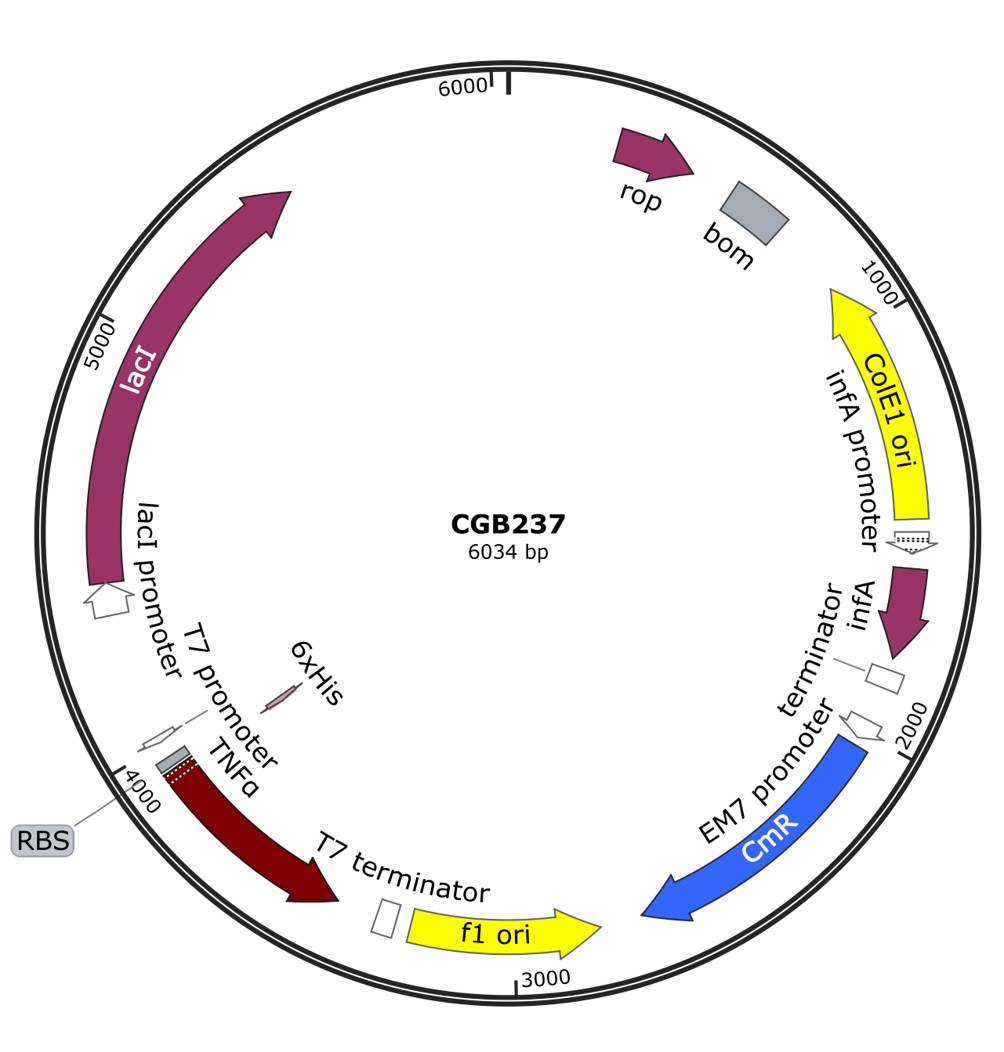

Supplement: Supplementary file 2 — SI Fig. 1: Plasmid map of CGB237 used for experiments shown in Fig. 4 and Fig. 7. [file 12934_2023_2291_MOESM2_ESM.jpg]
